# An Older People's Functional Mental Health Ward: A Year in Figures

**DOI:** 10.1192/bjo.2024.510

**Published:** 2024-08-01

**Authors:** Nicholas Rhodes, Andrew Donaldson, Kay Sunderland, Shaina Dillon, Patience Otaniyen

**Affiliations:** 1 NHS, Glasgow, United Kingdom; 2 NHS, Lanarkshire, United Kingdom; 3 NHS, Inverness, United Kingdom

## Abstract

**Aims:**

We aimed to review various health outcomes for patients admitted to an older adult psychiatry ward specialising in functional illness, over a one year period.

In 2020 the Mental Welfare Commission for Scotland highlighted a concern about the lack of evidence and data surrounding admission to older people’s functional mental health wards. We aimed to review this for North Lanarkshire and provide a comprehensive overview of our in-patient population that will aid in service review and improve care.

**Methods:**

We reviewed the electronic notes of all patients (total: 99) admitted to the ward over a one year period. Extracted data included demographics, medications, mental health act status, discharge destination and readmissions.

**Results:**

We found the average age was 73 years old and the median length of stay was 33 days (mean 63). Patients were admitted with a wide range of diagnosis including (most common to least): mood disorders, psychotic disorders, dementia, substance misuse and ARBD, delirium and personality disorders. 30% of patients required detention under the mental health act during their admission, but this fell to only 7% on discharge. 51% of patients were discharged on an antipsychotic. The majority of patients were discharged home; within a year 34% were readmitted to psychiatry and 40% required a medical admission.

**Conclusion:**

We found that our demographic information was broadly consistent with the mental welfare commission's findings. However there is a significant variation in length of stay shown by the difference in the mean and median, due to a small number of significantly longer admissions. Notably there were numerous admissions with a dementia as a primary diagnosis, on a functional ward. In this age group it was significant that a high proportion of patients were prescribed antipsychotics. Further work is required to better understand these findings.

